# The genome sequence of the Pine Hawkmoth, *Sphinx pinastri *(Linneaus 1758)

**DOI:** 10.12688/wellcomeopenres.19731.1

**Published:** 2023-07-20

**Authors:** Douglas Boyes, Clare Boyes, University of Oxford and Wytham Woods Genome Acquisition Lab, Darwin Tree of Life Barcoding collective, Wellcome Sanger Institute Tree of Life programme, Wellcome Sanger Institute Scientific Operations: DNA Pipelines collective, Tree of Life Core Informatics collective, Darwin Tree of Life Consortium

**Affiliations:** 1 UK Centre for Ecology & Hydrology, Wallingford, England, UK; 2 Independent researcher, Welshpool, Wales, UK

**Keywords:** Sphinx pinastri, Pine Hawkmoth, genome sequence, chromosomal, Lepidoptera

## Abstract

We present a genome assembly from an individual male *Sphinx pinastri* (the Pine Hawkmoth; Arthropoda; Insecta; Lepidoptera; Sphingidae). The genome sequence is 509.2 megabases in span. Most of the assembly is scaffolded into 28 chromosomal pseudomolecules, including the Z sex chromosome. The mitochondrial genome has also been assembled and is 15.3 kilobases in length.

## Species taxonomy

Eukaryota; Metazoa; Eumetazoa; Bilateria; Protostomia; Ecdysozoa; Panarthropoda; Arthropoda; Mandibulata; Pancrustacea; Hexapoda; Insecta; Dicondylia; Pterygota; Neoptera; Endopterygota; Amphiesmenoptera; Lepidoptera; Glossata; Neolepidoptera; Heteroneura; Ditrysia; Obtectomera; Bombycoidea; Sphingidae; Sphinginae; Sphingini; *Sphinx*; *Sphinx pinastri* (Linneaus 1758) (NCBI:txid987436).

## Background

The Pine Hawkmoth ( *Sphinx pinastri*) is a moth in the family Sphingidae found throughout Europe eastwards to the Balkans. The moth was accidentally introduced to North America and, in parts of its range, is a serious forest pest ( Leraut, 2006). The distribution of this moth increased in southern England during the 20th century, with a major spread occurring over the last 40 years ( Randle *et al.*, 2019). The increase in conifer plantations has aided the expansion of its range ( Heath & Emmet, 1963).

The Pine Hawkmoth is a large (forewing length 35–41 mm), rather plain, brownish-grey moth with longitudinal black streaks on its wings, which have chequered fringes. The thorax is bordered in black. In the UK, the moth is single-brooded, flying from May until early August ( Waring *et al.*, 2017). The moth lays its eggs in small groups on the needles of Scots pine and sometimes Norway spruce. The pupa is found either on, or just under, the ground, often under pine needles. The moth overwinters as a pupa for up to two years ( Heath & Emmet, 1963).

The Pine Hawkmoth sometimes comes to light, and feeds at flowers including the lesser butterfly orchid ( *Platanthera bifolia*) ( Steen, 2013). Research in Norway demonstrated that the timing of flower visits by the hawkmoth coincided with the release of terpenoids by the orchids. This strongly suggests that these chemicals play an important role in guiding the moth to the flowers ( Steen *et al.*, 2019).

A genome sequence from *S. pinastri* will be useful for comparative studies across the Lepidoptera. The genome of *S. pinastri* was sequenced as part of the Darwin Tree of Life Project, a collaborative effort to sequence all the named eukaryotic species in the Atlantic Archipelago of Britain and Ireland. Here we present a chromosomally complete genome sequence for *S. pinastri* based on a male specimen from Wytham Woods, Oxfordshire, UK.

## Genome sequence report

**Figure 1.**
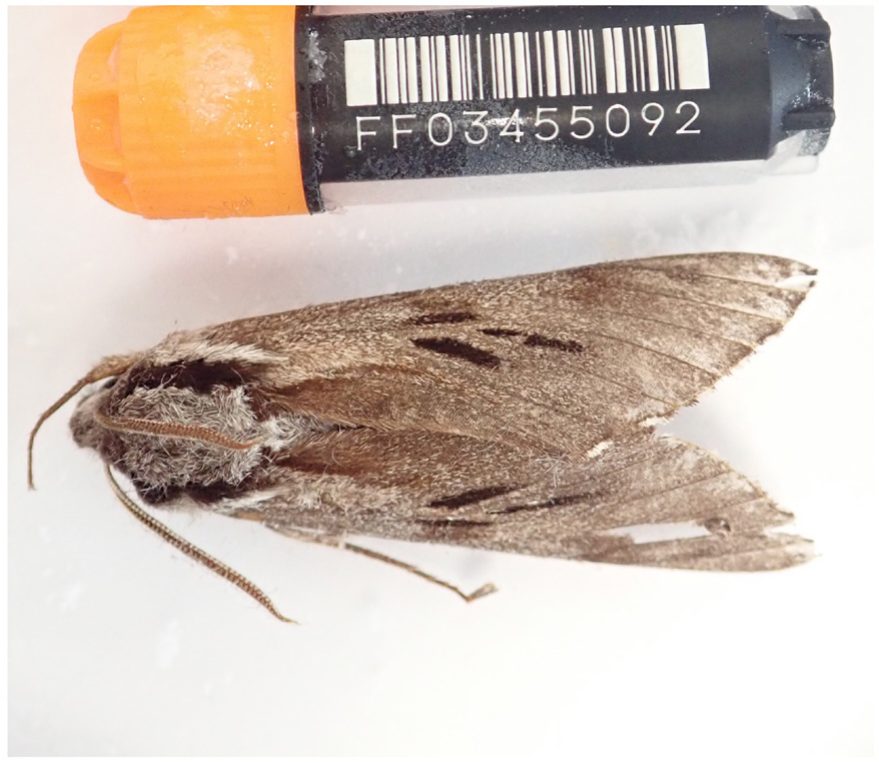
Photograph of the *Sphinx pinastri* (ilSphPina1) specimen used for genome sequencing.

The genome was sequenced from one male *Sphinx pinastri* ( Figure 1) collected from Wytham Woods, Oxfordshire, UK (51.77, –1.34). A total of 46-fold coverage in Pacific Biosciences single-molecule HiFi long reads was generated. Primary assembly contigs were scaffolded with chromosome conformation Hi-C data. Manual assembly curation corrected 9 missing joins or mis-joins and removed 5 haplotypic duplications, reducing the assembly length by 0.46% and the scaffold number by 6.38%.

**Figure 2.**
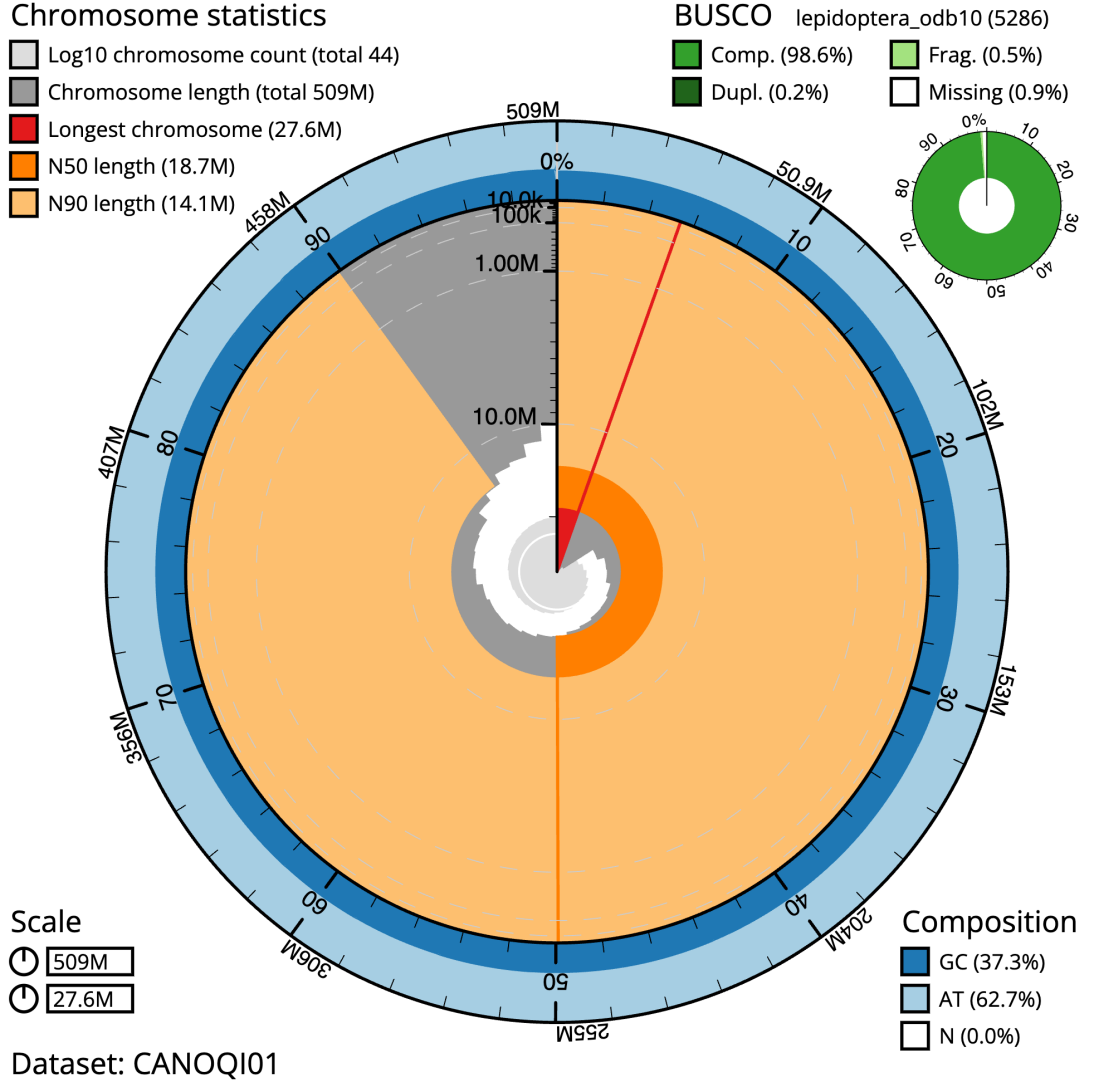
Genome assembly of *Sphinx pinastri*, ilSphPina1.1: metrics. The BlobToolKit Snailplot shows N50 metrics and BUSCO gene completeness. The main plot is divided into 1,000 size-ordered bins around the circumference with each bin representing 0.1% of the 509,238,608 bp assembly. The distribution of scaffold lengths is shown in dark grey with the plot radius scaled to the longest scaffold present in the assembly (27,562,872 bp, shown in red). Orange and pale-orange arcs show the N50 and N90 scaffold lengths (18,699,573 and 14,095,973 bp), respectively. The pale grey spiral shows the cumulative scaffold count on a log scale with white scale lines showing successive orders of magnitude. The blue and pale-blue area around the outside of the plot shows the distribution of GC, AT and N percentages in the same bins as the inner plot. A summary of complete, fragmented, duplicated and missing BUSCO genes in the lepidoptera_odb10 set is shown in the top right. An interactive version of this figure is available at https://blobtoolkit.genomehubs.org/view/ilSphPina1.1/dataset/CANOQI01/snail.

The final assembly has a total length of 509.2 Mb in 43 sequence scaffolds with a scaffold N50 of 18.7 Mb ( Table 1). Most (99.92%) of the assembly sequence was assigned to 28 chromosomal-level scaffolds, representing 27 autosomes and the Z sex chromosome. Chromosome-scale scaffolds confirmed by the Hi-C data are named in order of size ( Figure 2– Figure 5; Table 2). While not fully phased, the assembly deposited is of one haplotype. Contigs corresponding to the second haplotype have also been deposited. The mitochondrial genome was also assembled and can be found as a contig within the multifasta file of the genome submission.

**Table 1.** Genome data for *Sphinx pinastri*, ilSphPina1.1.

Project accession data		
Assembly identifier	ilSphPina1.1	
Species	*Sphinx pinastri*	
Specimen	ilSphPina1	
NCBI taxonomy ID	987436	
BioProject	PRJEB56126	
BioSample ID	SAMEA7701449	
Isolate information	ilSphPina1, male: abdomen (DNA sequencing), head (HiC sequencing), thorax (RNA sequencing)	
Assembly metrics *		*Benchmark*
Consensus quality (QV)	68.1	*≥ 50*
*k*-mer completeness	100%	*≥ 95%*
BUSCO **	C:98.6%[S:98.4%,D:0.2%], F:0.5%,M:0.9%,n:5,286	*C ≥ 95%*
Percentage of assembly mapped to chromosomes	99.92%	*≥ 95%*
Sex chromosomes	Z chromosome	*localised homologous pairs*
Organelles	Mitochondrial genome assembled	*complete single alleles*
Raw data accessions		
PacificBiosciences SEQUEL II	ERR10287559	
Hi-C Illumina	ERR10297841	
PolyA RNA-Seq Illumina	ERR10908606	
Genome assembly		
Assembly accession	GCA_947568825.1	
*Accession of alternate* *haplotype*	GCA_947568845.1	
Span (Mb)	509.2	
Number of contigs	52	
Contig N50 length (Mb)	17.7	
Number of scaffolds	44	
Scaffold N50 length (Mb)	18.7	
Longest scaffold (Mb)	27.6	

* Assembly metric benchmarks are adapted from column VGP-2020 of “Table 1: Proposed standards and metrics for defining genome assembly quality” from ( Rhie *et al.*, 2021). ** BUSCO scores based on the lepidoptera_odb10 BUSCO set using v5.3.2. C = complete [S = single copy, D = duplicated], F = fragmented, M = missing, n = number of orthologues in comparison. A full set of BUSCO scores is available at https://blobtoolkit.genomehubs.org/view/ilSphPina1.1/dataset/CANOQI01/busco.

**Figure 3.**
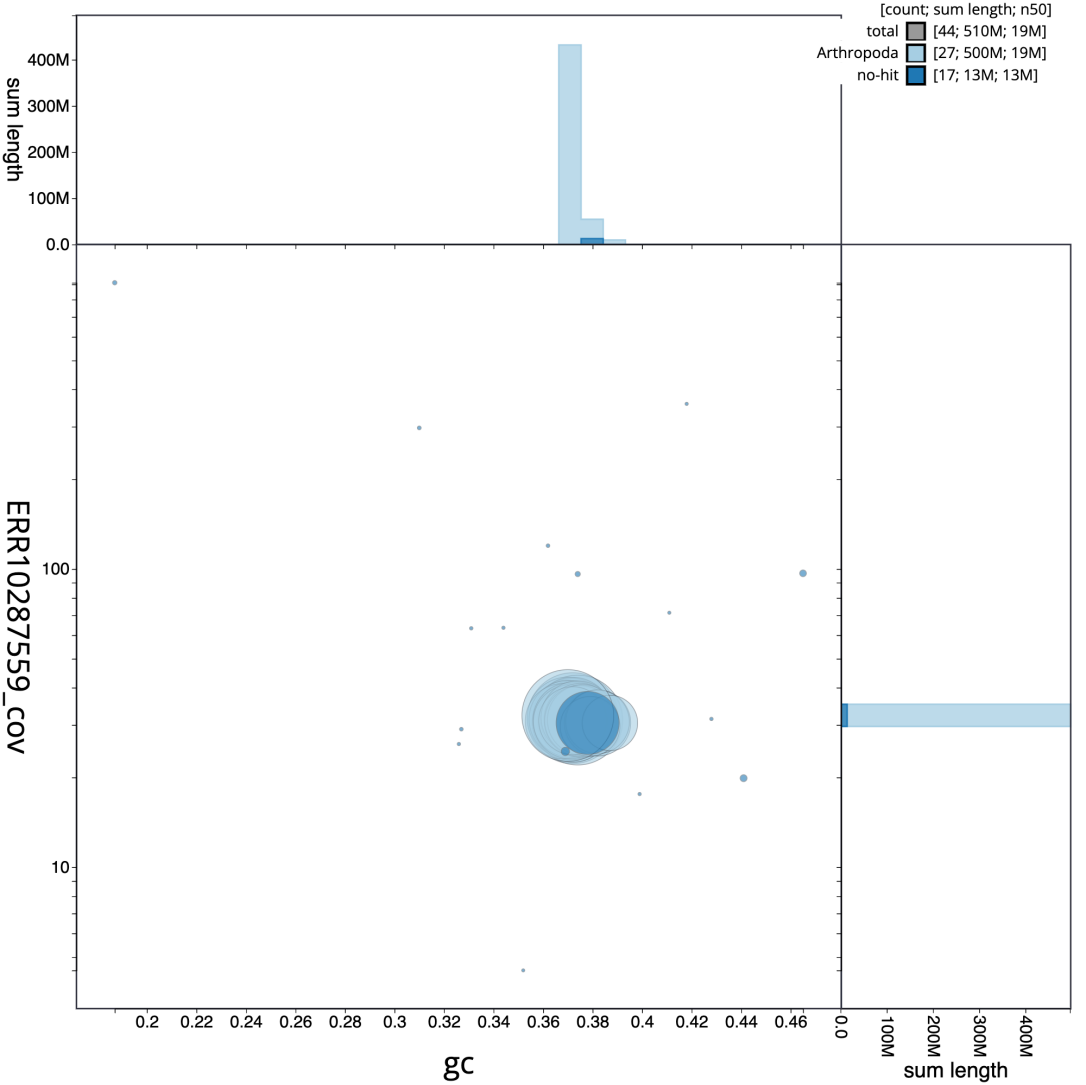
Genome assembly of *Sphinx pinastri*, ilSphPina1.1: BlobToolKit GC-coverage plot. Scaffolds are coloured by phylum. Circles are sized in proportion to scaffold length. Histograms show the distribution of scaffold length sum along each axis. An interactive version of this figure is available at https://blobtoolkit.genomehubs.org/view/ilSphPina1.1/dataset/CANOQI01/blob.

**Figure 4.**
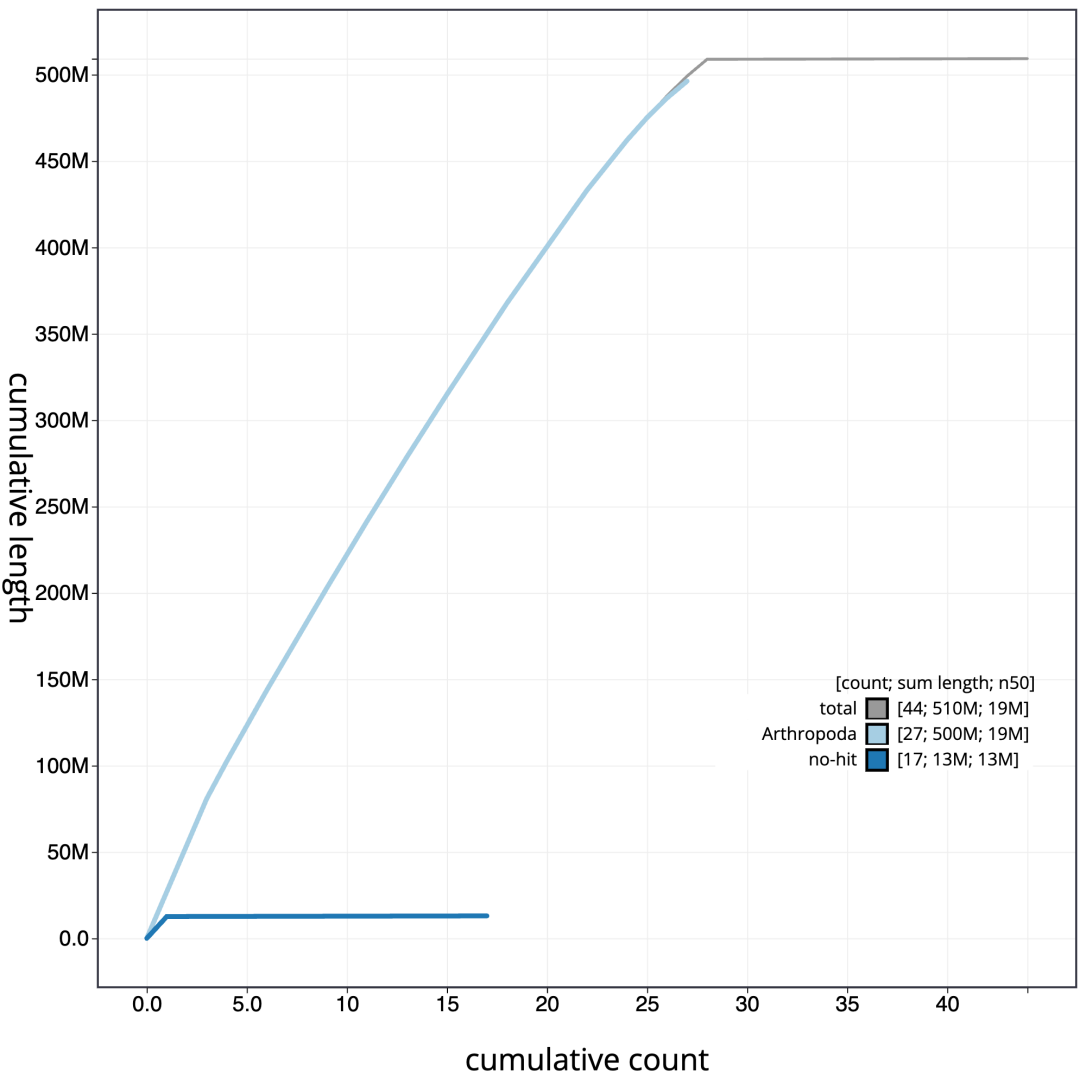
Genome assembly of *Sphinx pinastri*, ilSphPina1.1: BlobToolKit cumulative sequence plot. The grey line shows cumulative length for all scaffolds. Coloured lines show cumulative lengths of scaffolds assigned to each phylum using the buscogenes taxrule. An interactive version of this figure is available at https://blobtoolkit.genomehubs.org/view/ilSphPina1.1/dataset/CANOQI01/cumulative.

**Figure 5.**
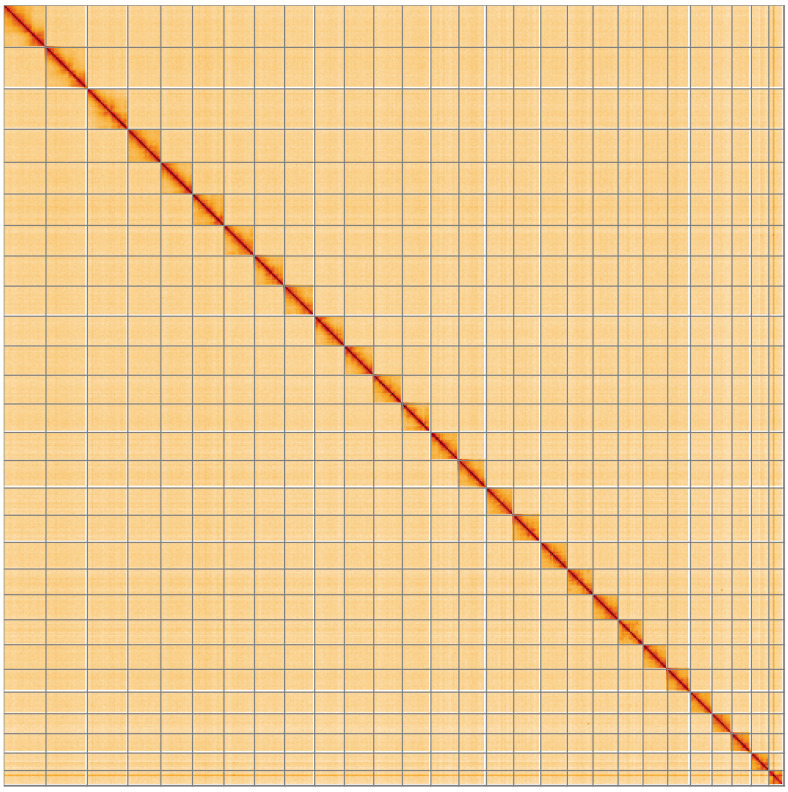
Genome assembly of *Sphinx pinastri*, ilSphPina1.1: Hi-C contact map of the ilSphPina1.1 assembly, visualised using HiGlass. Chromosomes are shown in order of size from left to right and top to bottom. An interactive version of this figure may be viewed at https://genome-note-higlass.tol.sanger.ac.uk/l/?d=X0FKgs1LRFu4Xc1NtTgp0Q.

**Table 2.** Chromosomal pseudomolecules in the genome assembly of *Sphinx pinastri*, ilSphPina1.

INSDC accession	Chromosome	Length (Mb)	GC%
OX387615.1	1	27.02	37.0
OX387616.1	2	26.4	37.5
OX387617.1	3	21.56	37.0
OX387618.1	4	20.62	37.0
OX387619.1	5	20.51	37.0
OX387620.1	6	19.91	37.5
OX387621.1	7	19.68	37.0
OX387622.1	8	19.62	37.0
OX387623.1	9	19.37	37.0
OX387624.1	10	19.12	37.0
OX387625.1	11	18.7	37.0
OX387626.1	12	18.61	37.5
OX387627.1	13	18.25	37.0
OX387628.1	14	17.99	37.0
OX387629.1	15	17.73	37.5
OX387630.1	16	17.68	37.0
OX387631.1	17	17.39	37.5
OX387632.1	18	16.71	37.5
OX387633.1	19	16.39	37.5
OX387634.1	20	16.24	37.5
OX387635.1	21	16.07	37.5
OX387636.1	22	14.86	37.0
OX387637.1	23	14.1	38.0
OX387638.1	24	13.02	38.0
OX387639.1	25	12.65	38.0
OX387640.1	26	11.39	38.0
OX387641.1	27	9.73	38.5
OX387614.1	Z	27.56	37.0
OX387642.1	MT	0.02	19.0

The estimated Quality Value (QV) of the final assembly is 68.1 with *k*-mer completeness of 100%, and the assembly has a BUSCO v5.3.2 completeness of 98.6% (single = 98.4%, duplicated = 0.2%), using the lepidoptera_odb10 reference set ( *n* = 5,286).

Metadata for specimens, spectral estimates, sequencing runs, contaminants and pre-curation assembly statistics can be found at https://links.tol.sanger.ac.uk/species/987436.

## Methods

### Sample acquisition and nucleic acid extraction

A male *Sphinx pinastri* (specimen ID Ox000585, individual ilSphPina1) was collected from Wytham Woods, Oxfordshire (biological vice-county Berkshire), UK (latitude 51.77, longitude –1.34) on 2020-07-05 using a light trap. The specimen was collected and identified by Douglas Boyes (University of Oxford) and preserved on dry ice.

DNA was extracted at the Tree of Life laboratory, Wellcome Sanger Institute (WSI). The ilSphPina1 sample was weighed and dissected on dry ice with tissue set aside for Hi-C sequencing. Abdomen tissue was cryogenically disrupted to a fine powder using a Covaris cryoPREP Automated Dry Pulveriser, receiving multiple impacts. High molecular weight (HMW) DNA was extracted using the Qiagen MagAttract HMW DNA extraction kit. HMW DNA was sheared into an average fragment size of 12–20 kb in a Megaruptor 3 system with speed setting 30. Sheared DNA was purified by solid-phase reversible immobilisation using AMPure PB beads with a 1.8X ratio of beads to sample to remove the shorter fragments and concentrate the DNA sample. The concentration of the sheared and purified DNA was assessed using a Nanodrop spectrophotometer and Qubit Fluorometer and Qubit dsDNA High Sensitivity Assay kit. Fragment size distribution was evaluated by running the sample on the FemtoPulse system.

RNA was extracted from thorax tissue of ilSphPina1 in the Tree of Life Laboratory at the WSI using TRIzol, according to the manufacturer’s instructions. RNA was then eluted in 50 μl RNAse-free water and its concentration assessed using a Nanodrop spectrophotometer and Qubit Fluorometer using the Qubit RNA Broad-Range (BR) Assay kit. Analysis of the integrity of the RNA was done using Agilent RNA 6000 Pico Kit and Eukaryotic Total RNA assay.

### Sequencing

Pacific Biosciences HiFi circular consensus DNA sequencing libraries were constructed according to the manufacturers’ instructions. Poly(A) RNA-Seq libraries were constructed using the NEB Ultra II RNA Library Prep kit. DNA and RNA sequencing was performed by the Scientific Operations core at the WSI on Pacific Biosciences SEQUEL II (HiFi) and Illumina NovaSeq 6000 (RNA-Seq) instruments. Hi-C data were also generated from head tissue of ilSphPina1 using the Arima2 kit and sequenced on the Illumina NovaSeq 6000 instrument.

### Genome assembly, curation and evaluation

Assembly was carried out with Hifiasm ( Cheng *et al.*, 2021) and haplotypic duplication was identified and removed with purge_dups ( Guan *et al.*, 2020). The assembly was then scaffolded with Hi-C data ( Rao *et al.*, 2014) using YaHS ( Zhou *et al.*, 2023). The assembly was checked for contamination and corrected as described previously ( Howe *et al.*, 2021). Manual curation was performed using HiGlass ( Kerpedjiev *et al.*, 2018) and Pretext ( Harry, 2022). The mitochondrial genome was assembled using MitoHiFi ( Uliano-Silva *et al.*, 2022), which runs MitoFinder ( Allio *et al.*, 2020) or MITOS ( Bernt *et al.*, 2013) and uses these annotations to select the final mitochondrial contig and to ensure the general quality of the sequence.

A Hi-C map for the final assembly was produced using bwa-mem2 ( Vasimuddin *et al.*, 2019) in the Cooler file format ( Abdennur & Mirny, 2020). To assess the assembly metrics, the *k*-mer completeness and QV consensus quality values were calculated in Merqury ( Rhie *et al.*, 2020). This work was done using Nextflow ( Di Tommaso *et al.*, 2017) DSL2 pipelines “sanger-tol/readmapping” ( Surana *et al.*, 2023a) and “sanger-tol/genomenote” ( Surana *et al.*, 2023b). The genome was analysed within the BlobToolKit environment ( Challis *et al.*, 2020) and BUSCO scores ( Manni *et al.*, 2021; Simão *et al.*, 2015) were calculated.

Table 3 contains a list of relevant software tool versions and sources.

**Table 3.** Software tools: versions and sources.

Software tool	Version	Source
BlobToolKit	4.0.7	https://github.com/blobtoolkit/blobtoolkit
BUSCO	5.3.2	https://gitlab.com/ezlab/busco
Hifiasm	0.16.1-r375	https://github.com/chhylp123/hifiasm
HiGlass	1.11.6	https://github.com/higlass/higlass
Merqury	MerquryFK	https://github.com/thegenemyers/MERQURY.FK
MitoHiFi	2	https://github.com/marcelauliano/MitoHiFi
PretextView	0.2	https://github.com/wtsi-hpag/PretextView
purge_dups	1.2.3	https://github.com/dfguan/purge_dups
sanger-tol/genomenote	v1.0	https://github.com/sanger-tol/genomenote
sanger-tol/readmapping	1.1.0	https://github.com/sanger-tol/readmapping/tree/1.1.0
YaHS	yahs-1.1.91eebc2	https://github.com/c-zhou/yahs

### Wellcome Sanger Institute – Legal and Governance

The materials that have contributed to this genome note have been supplied by a Darwin Tree of Life Partner. The submission of materials by a Darwin Tree of Life Partner is subject to the **‘Darwin Tree of Life Project Sampling Code of Practice’**, which can be found in full on the Darwin Tree of Life website here. By agreeing with and signing up to the Sampling Code of Practice, the Darwin Tree of Life Partner agrees they will meet the legal and ethical requirements and standards set out within this document in respect of all samples acquired for, and supplied to, the Darwin Tree of Life Project.

Further, the Wellcome Sanger Institute employs a process whereby due diligence is carried out proportionate to the nature of the materials themselves, and the circumstances under which they have been/are to be collected and provided for use. The purpose of this is to address and mitigate any potential legal and/or ethical implications of receipt and use of the materials as part of the research project, and to ensure that in doing so we align with best practice wherever possible. The overarching areas of consideration are:

- Ethical review of provenance and sourcing of the material
- Legality of collection, transfer and use (national and international)

Each transfer of samples is further undertaken according to a Research Collaboration Agreement or Material Transfer Agreement entered into by the Darwin Tree of Life Partner, Genome Research Limited (operating as the Wellcome Sanger Institute), and in some circumstances other Darwin Tree of Life collaborators.

## Data Availability

European Nucleotide Archive: *Sphinx pinastri* (pine hawkmoth). Accession number PRJEB56126; https://identifiers.org/ena.embl/PRJEB56126. ( Wellcome Sanger Institute, 2022) The genome sequence is released openly for reuse. The *Sphinx pinastri* genome sequencing initiative is part of the Darwin Tree of Life (DToL) project. All raw sequence data and the assembly have been deposited in INSDC databases. The genome will be annotated using available RNA-Seq data and presented through the Ensembl pipeline at the European Bioinformatics Institute. Raw data and assembly accession identifiers are reported in Table 1.

## Author information

Members of the University of Oxford and Wytham Woods Genome Acquisition Lab are listed here: https://doi.org/10.5281/zenodo.4789928.

Members of the Darwin Tree of Life Barcoding collective are listed here: https://doi.org/10.5281/zenodo.4893703.

Members of the Wellcome Sanger Institute Tree of Life programme are listed here: https://doi.org/10.5281/zenodo.4783585.

Members of Wellcome Sanger Institute Scientific Operations: DNA Pipelines collective are listed here: https://doi.org/10.5281/zenodo.4790455.

Members of the Tree of Life Core Informatics collective are listed here: https://doi.org/10.5281/zenodo.5013541.

Members of the Darwin Tree of Life Consortium are listed here: https://doi.org/10.5281/zenodo.4783558.
