# Supplementary material for: The genome sequence of the Pine Hawkmoth, Sphinx pinastri (Linneaus 1758)
Source: Wellcome Open Res. 2023 Jul 20;8:312. [Version 1] doi: 10.12688/wellcomeopenres.19731.1 (PMC10762287; doi:10.12688/wellcomeopenres.19731.1)
Supplement: Peer review history

# Reviewer report: Reviewer response for version 1

**DOI:** 10.21956/wellcomeopenres.21854.r71307

**Published:** 2024-01-08

**Authors:** Jorge Vieira

**Affiliations:** 1 Institute for Molecular and Cell Biology, Universidade do Porto, Porto, Portugal

This is a well written technically sound standard genome report. The analyses that have been performed show that the reported genome assembly is of high quality and that all efforts were made to produce the best possible genome assembly. Nevertheless, I only understood where the RNA sequencing data was used, when I read the Data availability section, as there is no indication elsewhere in the report that there was an attempt to produce a genome annotation.

Are sufficient details of methods and materials provided to allow replication by others?

Yes

Is the rationale for creating the dataset(s) clearly described?

Yes

Are the datasets clearly presented in a useable and accessible format?

Yes

Are the protocols appropriate and is the work technically sound?

Yes

Reviewer Expertise:

Genomics, Transcriptomics, Evolutionary Biology, Population Genetics.

I confirm that I have read this submission and believe that I have an appropriate level of expertise to confirm that it is of an acceptable scientific standard.

# Reviewer report: Reviewer response for version 1

**DOI:** 10.21956/wellcomeopenres.21854.r71305

**Published:** 2024-01-02

**Authors:** Ljiljana Šašić Zorić

**Affiliations:** 1 BioSense Institute, University of Novi Sad, Novi Sad, Serbia

“The genome sequence of the Pine Hawkmoth, Sphinx pinastri (Linneaus 1758)” is a technical report on the genome assembly of the forest pest species, Pine Hawkmoth. The sequencing approach included Pacific Biosciences HiFi sequencing and scaffolding with chromosome conformation using Hi-C data. Additionally, RNA sequencing was also done.

 Methodology is well described, and genome assembly data is clearly presented. There is no thorough analysis on RNA-Seq data, only raw data accession. However, authors stated that RNA-Seq data will be used for genome annotation and provided through Ensembl pipeline (European Bioinformatics Institute).

 A genome sequence of the Pine Hawkmoth will be useful for new marker development as well as comparative studies across Lepidoptera.

 Suggestions for correction:

 In the section “Genome sequence report” number of sequence scaffolds is 43, while in Table1 is 44.

Are sufficient details of methods and materials provided to allow replication by others?

Yes

Is the rationale for creating the dataset(s) clearly described?

Yes

Are the datasets clearly presented in a useable and accessible format?

Yes

Are the protocols appropriate and is the work technically sound?

Yes

Reviewer Expertise:

Population genetics and molecular taxonomy

I confirm that I have read this submission and believe that I have an appropriate level of expertise to confirm that it is of an acceptable scientific standard.

# Reviewer report: Reviewer response for version 1

**DOI:** 10.21956/wellcomeopenres.21854.r69541

**Published:** 2023-11-21

**Authors:** Marko Mutanen

**Affiliations:** 1 University of Oulu, Oulu, Finland

This study publishes a reference genome for a moth of the family Sphingidae, *Sphinx pinasti* (Lepidoptera). The species is widespread and common in Europe, and it represents a species of the type genus of the family. The article does not summarize how many members of the family are with reference genomes generated before, and what is the closest relatives with genome available. The manuscript would benefit from such a short overview.

 The genome is chromosomal-level, and the quality measures all indicate that it is of high quality and reliability. There is very little missing data and for example very large proportion of Lepidoptera BUSCO markers were mapped. The work is therefore technically sound.

 While there is no doubt about the correct identification in this case, I find that it would be a good practise to validate the identification through DNA barcode region comparison as the assembly includes also the full mitochondrial genome. In some cases this might reveal that the sequenced genome does not represent the species it was believed to represent.

 This work is an important contribution which helps us to understand e.g. Lepidoptera phylogenomics, but is useful in various other comparative settings.

Are sufficient details of methods and materials provided to allow replication by others?

Yes

Is the rationale for creating the dataset(s) clearly described?

Yes

Are the datasets clearly presented in a useable and accessible format?

Yes

Are the protocols appropriate and is the work technically sound?

Yes

Reviewer Expertise:

I am an expert of Lepidoptera systematic and taxonomy, including molecular tools. I have a plenty of experience of genomic methods, but little about genome assembly. I do not have personal experience of several of the used software.

I confirm that I have read this submission and believe that I have an appropriate level of expertise to confirm that it is of an acceptable scientific standard.
